# Extracellular Vesicles: How Drug and Pathology Interfere With Their Biogenesis and Function

**DOI:** 10.3389/fphys.2018.01394

**Published:** 2018-10-01

**Authors:** Daniela Cesselli, Pietro Parisse, Aneta Aleksova, Claudia Veneziano, Celeste Cervellin, Andrea Zanello, Antonio Paolo Beltrami

**Affiliations:** ^1^Department of Medicine, University of Udine, Udine, Italy; ^2^Elettra-Sincrotrone Trieste S.C.p.A., Trieste, Italy; ^3^Cardiovascular Department, Azienda Sanitaria Universitaria Integrata di Trieste – University of Trieste, Trieste, Italy

**Keywords:** extracellular vesicles, exosomes, non-coding RNA, miRNA, heart failure, pharmacological therapy

## Abstract

Extracellular vesicles (EV) are at the center of an intense activity of investigation, both for their possible employment as biomarkers of ongoing pathologic processes and for their broad range of biological activities. EV can promote tissue repair in very different pathologic settings, including hindlimb and myocardial ischemia. Importantly, the exact mode of action of EV is still partly understood, since they may act by modulating growth factors and cytokines, signaling pathways, and by transferring non-coding RNAs to target cells. However, the term EV identifies cell derived, enveloped particles very heterogeneous in size, composition, and biogenesis. Therefore, part of the controversies on the biological effects exerted by EV is a consequence of differences in methods of separation that result in the enrichment of different entities. Since technical challenges still hamper the highly specific sorting of different EV subpopulations, up to now only few investigators have tried to verify differences in the biological effects of specific EV subtypes. This review summarizes the current state of the art on the comprehension of mechanisms involved in EV biogenesis and release, which is a prerequisite for understanding and investigating the impact that pathology and drug therapy may exert on the secretion and composition of EV. Finally, we described both the mechanism involved in the modulation of EV secretion by drugs commonly used in patients affected by heart failure, and how pathophysiological mechanisms involved in heart disease modify EV secretion.

## Introduction

Extracellular vesicles (EV) are gaining momentum as potential biomarkers and for their broad range of biological activities. Indeed, a large body of literature, generated by independent laboratories, has shown, over the last few years, that EV can promote tissue repair in very different pathologic settings, ranging from cutaneous wound healing and bone regeneration to hindlimb and myocardial ischemia. Consistently, it is now generally accepted that stem cells and cell based therapies act, at least in part, through the release of EV (see Chen et al., 2017). However, the exact mode of action of EV is still object of intense investigation. Indeed, EV may act by modulating growth factors and cytokines (e.g., IGF, SDF1, NGF, and HGF), signaling pathways (e.g., Akt, ERK, Wnt/β-catenin, Hippo/YAP, and STAT3), and miRNA (e.g., miR-21, -23a, -125b, and -145) (Shabbir et al., 2015; Zhang et al., 2016; Fatima et al., 2017). Moreover, the coordinated action of these EV components has been shown to regulate stem cell proliferation and differentiation, stimulate angiogenesis, regulate epithelial to mesenchymal transition, reduce fibrosis and apoptosis (reviewed in Fatima et al., 2017).

Extracellular vesicles play a relevant role in the regulation of immunity, where they promote both immune stimulation and suppression (see Robbins and Morelli, 2014 for review), in a broad range of physiological and pathological conditions. With regard to immune stimulation, EV can both trigger T cell activation, acting directly as antigen-presenting vesicles, and elicit an indirect, dendritic cell (DC)-mediated, effect (Nolte-’t Hoen et al., 2009; Robbins and Morelli, 2014). Conversely, the mechanisms of the immunosuppressive function of EV, which have been experimentally exploited in the cardiac transplantation field to reduce cardiac allograft rejection, are still under scrutiny (Peche et al., 2003; Robbins and Morelli, 2014).

Given their complex mechanism of action and broad effects, EV are also involved in the pathogenesis of distinct pathological conditions, including inflammatory, autoimmune, and infectious diseases (Robbins and Morelli, 2014) as well as neurodegenerative or prion-related diseases, where they may enhance the spreading of toxic proteins or aggregates through the brain (Guo et al., 2016; Xiao T. et al., 2017).

Nonetheless, the term EV is a very general one that embraces cell derived, enveloped particles very heterogeneous in size, composition, and biogenesis. Therefore, part of the controversies on the biological effects exerted by EV is a consequence of differences in methods of separation that result in the enrichment of different entities. Specifically, EV have been divided into broad categories, mainly depending on their size and biogenesis (i.e., microvesicles (MVs), apoptotic bodies, and exosomes). While the first two bud from the plasma membrane, exosomes derive from multivesicular bodies (MVB) (Maas et al., 2017). Importantly, although both MVs and exosomes can transfer their cargo to recipient cells, influencing their behavior (Antonyak and Cerione, 2015), a large body of recent literature is focusing on exosomes, the smallest EV. However, only few investigators have tried to verify differences in the biological effects of these subtypes, possibly as a consequence of the difficulties in sorting with high specificity the different EV populations (Kanada et al., 2015).

Aim of this review is to discuss the impact that drugs and comorbidities exert on the biogenesis and function of EV, with particular reference to the cardiovascular system.

## Exosomes and Microvesicles: What Are Them?

Although cell derived vesicles have been described in the mid 20^th^ century, their biological function was initially only marginally understood, and they were considered to be mostly “cell dust” or a system of “trash disposal” (Laberge et al., 2018). It was only recently that the role played by EV as a primary mechanism of intercellular communication has emerged, mainly for their ability to transfer macromolecules, including proteins and RNA, among cells (Valadi et al., 2007; Al-Nedawi et al., 2009).

As anticipated, the term EV comprises different entities that differ in size, composition, and biogenesis. Specifically, three broad categories have been identified: exosomes, that are ≈30–100 nm in size and originate intracellularly from the MVB; MVs (also named ectosomes or microparticles), whose size ranges from ≈200 to 1,000 nm and arise via a direct budding of the plasma membrane; and apoptotic bodies, that are 1,000–5,000 nm in size and are generated as a consequence of programmed cell death. In recent years, it has become apparent that the picture is more complex than previously thought and that the overlap in size distribution among different EV types impairs our ability to separate them merely on physical basis (Tkach et al., 2018). Indeed, according to opinion leaders, the most popular methods of exosome purification (i.e., ultracentrifugation, 200 nm filtration, and precipitation) actually co-isolate different types of EV (Tkach and Thery, 2016).

## Exosome Biogenesis and Sorting of Exosomal Cargo

Concerning the biogenesis of EV, it involves, for every subtype, the negative curvature of cellular membranes (i.e., bending the membrane away from the cytoplasm). While exosomes are first released within the MVB as intraluminal vesicles (ILV), larger EV are directly pinched off the plasma membrane. The negative curvature of the cellular membranes can be initiated by different mechanisms, that are divided into endosomal sorting complex required for transport (ESCRT)-dependent and ESCRT-independent.

### Endosomal Sorting Complex Required for Transport, ESCRT

In the early 2000’s, a molecular machinery responsible for the negative bending of cell membranes (i.e., ESCRT) was identified. It comprises about 30 proteins, assembled in five functional units that are required for: cargo recognition (ESCRT-0), its sequestration into endosomal microdomains (ESCRT-I and II), the constriction of the neck of the nascent vesicle (ESCRT-III), and finally, membrane scission and ESCRT disassembly (promoted by the Vps4 complex) (Furthauer, 2018). A further support to membrane budding is provided by the interaction of syndecans and sintenin with Alix, an auxiliary component of the ESCRT complex (Baietti et al., 2012). In this pathway, that is particularly relevant for the regulation of the turnover of membrane proteins, ubiquitination of transmembrane proteins is required for their recognition and sorting by ESCRT-0, -I, and -II complexes (Urbanelli et al., 2013; Colombo et al., 2014).

However, other sorting mechanisms are in place too. Specifically, cytosolic proteins can be recruited to late endosomes by a microautophagy-like mechanism, which is distinct from chaperone-mediated autophagy, and requires hsc70, and ESCRT-I and -III (Sahu et al., 2011). Importantly, several molecules, including the pigment cell-specific type I integral membrane protein PMEL and CD63 in melanocytes (van Niel et al., 2011), and the class II major histocompatibility complex (MHC) in DCs (Buschow et al., 2009), can be inserted into ILV following ESCRT independent pathways.

### Lipid Dependent Mechanisms

Ceramide production induced by neutral sphingomyelinase 2 (nSMase 2) is an important ESCRT-independent mechanism involved in ILV formation, possibly as a consequence of the ability of members of this family of lipids to cause membrane curvature (Trajkovic et al., 2008). However, the hypothesis that ceramide acts only by bending the membrane may be too simplistic (Kajimoto et al., 2013). Indeed, ceramide is at the center of the sphingolipid metabolism, where it acts as either a product or a precursor for several metabolites, including sphingosine-1-phosphate (S1P). Many of these metabolites have complex biological effects that are usually referred to as “sphingolipid rheostat” (see discussion later) (Newton et al., 2015). Specifically, it has been recently shown that the continuous activation of the inhibitory G protein (Gi) coupled S1P receptors on MVB is required for cargo sorting into ILV destined to the exosomal pathway. Conversely, inhibition of S1P production reduces CD63, CD81, and flotillin in ILV (Kajimoto et al., 2013). Intriguingly, a reciprocal role for ceramide and S1P on autophagy induction and mTOR pathway modulation has been described (Taniguchi et al., 2012). This finding is consistent with the concept that exosome secretion and the autophagy lysosome pathway are coordinated mechanisms and is reinforced by the observation that lysosome inhibition increases EV release (Baixauli et al., 2014; Miao et al., 2015). How these two different fates are regulated is still an area of investigation. Indeed, MVB are not a homogenous population, and it has been suggested that different subpopulations of MVB exist, with some (e.g., those rich in cholesterol) that are targeted for exocytosis.

Another lipid-dependent mechanism that is involved in the biogenesis of exosomes relies on the activity of phospholipase D (PLD). Upon stimulation with ionomycin, this enzyme is relocated from the plasma membrane to intracellular compartments and its activity correlates with the extent of exosome secretion (Laulagnier et al., 2004).

Furthermore, both PLD and phospholipase C (PLC) are involved in the production of diacylglycerol (DAG), which regulates several checkpoints of the vesicle secretory pathway (Alonso et al., 2011; Mazzeo et al., 2016). Diacylglycerol kinase (DGK) can convert DAG into phosphatidic acid, which can induce a negative membrane curvature and is a precursor of bis(diacylglycero)phosphate (BDP) (Kooijman et al., 2003). The latter or other substrates could be converted into Lysobisphosphatidic acid (LBPA) or bis(monoacylglycero)phosphate (BMP) by phospholipase A2 (PLA2) (Amidon et al., 1996). BMP, which increases its concentration in late endosomes/MVB (Subra et al., 2007), promotes the formation of ILV-like structures at low pH, interacting with Alix (Matsuo et al., 2004). Finally, BMP binds the chaperone Hsp70 (Kirkegaard et al., 2010) and seems to be critically involved in regulating the levels of cholesterol transported in endosomes (Chevallier et al., 2008).

### Tetraspanins

An additional, ESCRT-independent mechanism of ILV formation is provided by proteins of the tetraspanin family (that include CD9, CD63, CD81, CD82, and CD151). An increasing amount of evidence supports this notion, as suggested by both: the ability of cells in which the ESCRT has been silenced to release CD63 positive EV, and the reduced ability of DCs of CD9 knockout mice to promote exosome secretion (Stuffers et al., 2009; Chairoungdua et al., 2010; Andreu and Yanez-Mo, 2014). Tetraspanins are palmytoilated proteins characterized by four transmembrane domains and the ability to organize membrane domains (named tetraspanins-enriched domains, TEM), clustering and interacting with a large number of distinct transmembrane and cytosolic proteins, cholesterol and gangliosides (Andreu and Yanez-Mo, 2014). They can regulate membrane shape (Grove, 2014), mostly promoting (or inhibiting, as in the case of CD82) membrane bending and regulating actin polymerization (Bari et al., 2011). Additionally, tetraspanins have been involved in sorting the intracellular cargo toward EV. Specifically, β-catenin, Wnt11, and PMEL, as well as matrix metalloproteases, such as CD10, and MHC-I and -II molecules have been shown to be sent to EV in a tetraspanin dependent fashion (Andreu and Yanez-Mo, 2014). Furthermore, by studying the interactome of EV pulled down employing antibodies against the C-terminal regions of both tetraspanins (CD9, CD81, and CD151) and tetraspanin-associated immunoglobulin superfamily receptors (ICAM-1, VCAM-1, and EWI-2), it was shown that ≈45% of the proteins annotated as exosomal indeed were part of the interactome of CD81 and/or EWI-2 (Perez-Hernandez et al., 2013).

### RNA Sorting Mechanisms

A special mention deserves the mechanism that regulate RNA sorting into EV. Indeed, several studies confirmed that the RNA content of parental cells is significantly different from that of cell-derived vesicles, pointing to a selective sorting of target RNA species to the EV (Ratajczak et al., 2006; Skog et al., 2008; Nolte-’t Hoen et al., 2012). Deep sequencing experiments performed on DC derived EV have shown that the majority of extracellular RNA consists of small (<200 nucleotides) RNAs. Most of these are non-coding RNAs, distinct from microRNA and large intervening non-coding RNAs, and represent transcripts or cleavage products overlapping with protein coding regions, repeat sequences, or structural RNAs. It has been suggested that some of these could play a role in the specific sorting of regulatory RNAs into the EV (Nolte-’t Hoen et al., 2012). A broader picture was obtained by analyzing the extracellular RNA released by glioblastoma stem cells. In this setting, authors have shown that MV RNA content is more similar to that of the parent cell, while the exosomal one is more distinct (Wei et al., 2017). With regard to miRNA, bioinformatics and mutagenesis analyses have demonstrated that EXOmotifs regulate both their binding to hnRNPA2B1 and their loading into exosomes, upon sumoylation (Villarroya-Beltri et al., 2013). Additionally, Annexin-2, a protein that is found with high abundance in exosomes, binds miRNA in a Ca^2+^ dependent fashion. The role played by this protein in sorting miRNA into exosomes is provided by silencing experiments that showed a decrease in the amount of miRNA loaded into exosomes, without modifying their expression profile (Hagiwara et al., 2015). It has been additionally suggested that selective sorting of RNAs to EV could depend on their affinity to ceramide and sphingosine rich raft like structures present in MVB (Kosaka et al., 2010; Janas et al., 2012, 2015). This property may be either mediated by binding of target RNA to hnRNPA2B1 or consequent to hydrophobicity given directly by specific RNA sequences or by enzymatic modifications, such as miRNA methylation or tRNA isoprenylation (Janas et al., 2012, 2015).

## Microvesicle Biogenesis

Somewhat less understood and investigated are the mechanisms that regulate plasma membrane budding and MV biogenesis. While, as for exosomes, the ESCRT complex is involved in the biogenesis of MV, MV production and release can also be induced by calcium signaling and ATP and lipid mediated mechanisms. Exosomes and MVs differ also in the mechanisms involved in protein and RNA sorting.

### ESCRT

Although the biogenesis of exosomes and that of MVs are distinct processes, they both share part of the same molecular mechanisms. Indeed, one component of ESCRT-I (i.e., TSG101), that is hijacked by HIV to promote the release of viral particles, can be recruited to the plasma membrane by arrestin domain-containing protein 1 (ARRDC1) to promote, together with VPS4, MV release (Nabhan et al., 2012). Importantly, these ARRDC1-mediated MVs lack the endosomal markers CD63 and LAMP1 (Nabhan et al., 2012).

Additionally, it has been shown that ESCRT components can be found in the plasma membrane too, where they can form oligomeric complexes (Welsch et al., 2006). However, the ability of these molecules to promote the formation of MVs with the same mechanism that is observed in ILV biogenesis is uncertain (Yang and Gould, 2013).

### Calcium Mediated Processes

Increased intracytoplasmic calcium levels, either employed as second messenger of different signal transduction pathways or occurring as a consequence of cell apoptosis and necrosis, can activate calcium sensitive proteins involved in MV biogenesis. Specifically, gelsolin, a ubiquitous actin binding protein that can remodel the actin cytoskeleton (Li et al., 2012) and calpain, a family of cysteine protease that modulate the function of their substrates by performing “limited proteolysis” (Ono et al., 2016), can act in synergy to promote cytoskeleton detachment and membrane blebbing (Miyoshi et al., 1996). Moreover, the activities of enzymes involved in regulating plasma membrane asymmetry, such as aminophospholipid translocase (that transports phosphatidylserine and phosphatidylethanolamine from the outer layer to the inner layer) and lipid scramblase (that promotes the movement of lipids across the cell membrane) are modulated in opposite fashions by calcium, resulting in loss of plasma membrane asymmetry and exposure of phosphatidylserine (Piccin et al., 2007). Furthermore, Annexin-2, a protein involved in calcium induced exocytosis has been also shown to promote MV release (Zhang W. et al., 2013).

### ATP and Lipid Mediated Mechanisms

Upon stimulation of P2X_7_ purinergic receptors by ATP released in the extracellular space, the acid sphingomyelinase is rapidly activated and translocated to the outer layer of the plasma membrane, where it cleaves sphingomyelin, increasing the efflux of cholesterol and membrane fluidity, thus facilitating membrane shedding (Bianco et al., 2009).

### Membrane Targeting and Protein Crowding

Protein sorting to nascent EV is thought to depend on the presence of anchors [i.e., protein myristoylation, phosphatidylinositol-4,5-bisphosphate (PIP2)-binding domains, phosphatidylinositol-(3,4,5)-trisphosphate-binding domains] that promote their association with the plasma membrane compartment (Shen et al., 2011). However, a second signal provided by the higher-order oligomerization of surface proteins seems to be required for their secretion within MV. Intriguingly, the presence of an endosomal targeting domain was not efficient in promoting protein secretion via EV, in this experimental model (Shen et al., 2011). A corollary to this observation is that membrane targeted proteins that form higher-order oligomeric complexes can be secreted into EV. Indeed, tetraspanins, that can be found as TEM also in the plasma membrane, can interact with the cytoskeleton via ezrin/radixin/moesin proteins, and can be thus released into MVs (Sala-Valdes et al., 2006; Yang and Gould, 2013; Andreu and Yanez-Mo, 2014).

Last, protein lateral confining and crowding mechanisms can bend membranes and may provide a biophysical explanation for EV formation (Stachowiak et al., 2012; Derganc and Copic, 2016).

### RNA Sorting Mechanisms

As opposed to what was shown for exosomes, results obtained with next-generation sequencing suggest that the selection of the cargo may be, in this case, less stringent. Indeed, the RNA content of MV more closely resembles the parental cell one (Wei et al., 2017).

## Secretion of Exosome and MV

A relevant question in EV biology is whether their secretion is constitutive or regulated. By understanding the mechanisms that regulate the secretion of EV, we could gain better insights into how ischemia and pharmacological therapies may impact on their release.

### Regulation of Exosome Release

Once formed, ILV are released into the extracellular space, following the fusion of MVB or endosomal membranes with the plasma membrane, with a process that is regulated by the same mechanisms that control the trafficking of carrier vesicles. Specifically, transport, docking and fusion of vesicles to the plasma membrane are governed by the coordinated action of the cytoskeleton, of small GTPases of the Rab family, tethering factors, and soluble N-ethylmaleimide-sensitive factor attachment proteins -SNAP- and their receptors -SNARE-. More than 60 Rab proteins are encoded by the human genome. Each family member has a peculiar subcellular localization and is involved in distinct intracellular transport steps (Wandinger-Ness and Zerial, 2014). Of these, only Rab11, Rab27, Rab35, and possibly Rab22a, are validated regulators of exosome release (Colombo et al., 2014; Wang T. et al., 2014). Intriguingly, since Rab35, Rab11, and Rab27 are associated with early, recycling, and late endosomes, respectively, it has been hypothesized that they regulate the secretion of different subsets of exosomes. Differing in their origin, these subsets of exosomes are expected to express distinct markers, reflecting their sub cellular source (Colombo et al., 2014). Noteworthy, Rab proteins not directly involved in the exosome secretory pathways can be found within the vesicles, potentially reflecting a mechanism for the regulation of the correct intracellular levels and sub cellular localization of specific isoforms (Blanc and Vidal, 2017). With regard to the mechanisms regulating membrane fusion, SNAP and SNARE proteins, such as YKT6, VAMP7, VAMP3, and syntaxin-1a have been shown to be crucial for this process (Fader et al., 2009; Gross et al., 2012; Koles et al., 2012).

Exosomes can be released both in a constitutive and in a regulated fashion. With regard to upstream regulators of exosome secretion, it has been shown that the interaction between DCs and antigen specific CD4^+^ T lymphocytes can stimulate exosome release. Other triggers are represented by increased intracellular calcium levels, depolarization or neurotransmitter stimulation of neurons and glial cells (Colombo et al., 2014). Moreover, stressors able to induce p-53 can promote exosome secretion by upregulating the expression of its target gene TSAP6 (alias STEAP3), a multiple transmembrane protein involved both in iron homeostasis and Toll Like Receptor 4 mediated signaling (Lespagnol et al., 2008; Zhang et al., 2012).

Although considered to be a primary regulator of MV release, ADP ribosylation factor 6 (ARF6) promotes exosome secretion via its effector PLD2, an enzyme that regulates the cellular levels of phosphatidic acid, interacting with syntenin and Alix (Ghossoub et al., 2014). In line, DGK too, that metabolizes DAG to phosphatidic acid, promotes exosome secretion, in T cells (Alonso et al., 2011).

Other mechanisms of modulation of exosome secretion that have been described more recently are mediated by purinergic receptors, heparanase and ISGylation. The first ones (specifically P2X_7_ or P2Y12), are stimulated by extracellular ATP, and have been recently recognized as a relevant stimulus for exosome release (Drago et al., 2017). The second one is an enzyme that promotes angiogenesis and metastatic spread in many cancers, and can regulate the syntenin/Alix pathway by tailoring syndecans (Roucourt et al., 2015). ISGylation, instead, describes a mechanism of post-translational protein modification that leads to the covalent binding of target proteins to ISG15, an interferon induced ubiquitin-like protein. Recent data indicate that Interferon-I may reduce exosome secretion by promoting the ISGylation and degradation of TSG101 (Villarroya-Beltri et al., 2016).

Last, hypoxia is a prominent inducer of exosome release via HIF1α/HIF2α, both in the cancer and non-neoplastic settings (King et al., 2012; Kucharzewska et al., 2013; Zhang W. et al., 2017; Panigrahi et al., 2018).

### Regulation of MV Release

While exosomes have been studied mainly in unstimulated cells, MV have been mostly analyzed following the application of specific stimuli (Colombo et al., 2014). However, recent data indicate that both mechanisms of constitutive and stimulated MV release exist, although the composition of MV released in these two different conditions can change (Drago et al., 2017).

Platelet derived MV (pMV) deserve a special mention, since they are the most abundant MV found in the circulation and exert procoagulant and proinflammatory functions (Badimon et al., 2016). Formation of pMV can be induced by a large number of stimuli, some of which shared with other cell types. Exercise training, heat stress, shear stress, cyclic strain, cardiac stress have all been shown to elicit pMV and MV release (Miyazaki et al., 1996; Augustine et al., 2014; Letsiou et al., 2015; Wilhelm et al., 2017). Moreover, different agonists have the ability to promote the release of MV. Among these, we should mention ATP or ADP stimulation, possibly in the presence of lipopolysaccharide priming, acting on either P2X_7_ or P2Y_12_ purinergic receptors, can lead to a raise in the intracellular Ca^2+^ levels, promoting cytoskeleton remodeling and MV release (Kahner et al., 2008; Bianco et al., 2009; Gulinelli et al., 2012; Colombo et al., 2014; Takenouchi et al., 2015; Drago et al., 2017). Furthermore, other proinflammatory (e.g., TNFα) and procoagulant factors (e.g., thrombin), together with reactive oxygen species can all induce MV release (Sapet et al., 2006; Szotowski et al., 2007). Last, hypoxia, via HIF1α- and HIF2α-induced Rab22a expression, has been associated with MV secretion (Wang T. et al., 2014). However, many variables can influence the response to the same stimulus, such as the cell type, the subject gender, age, and comorbidities.

## Impact of Drugs and Diseases on Exosome Release

As anticipated, drugs and comorbidities can influence the mechanisms of biogenesis and release of exosomes and MV, thus influencing this mechanism of intercellular communication.

### Cardiovascular Diseases and Comorbidities

**Table 1** summarizes evidence of the association between the release of EV (both MVs and exosomes) and: cardiovascular risk factors (e.g., diabetes, hypertension, and sleep apnea), atherosclerosis and its complications. Furthermore, both the cell types involved in EV release and target cells are shown.

**Table 1:** Evidence of EV release in pathologic conditions.

*Pathological condition*	Type of EV released	Releasing cell	Known mechanism of release	Effect
*Type 1 diabetes*	Microparticles	Endothelial cells (Jansen et al., 2013a), platelets (Ishida et al., 2016), leukocytes, SMC (Chiva-Blanch et al., 2016), and podocytes (Lytvyn et al., 2017; Munkonda et al., 2018).	Hyperglycemia (Jansen et al., 2013a; Lytvyn et al., 2017).	Endothelial dysfunction (Jansen et al., 2013a; Ishida et al., 2016), procoagulant effect (Sabatier et al., 2002), inflammation (Jansen et al., 2013a), and kidney fibrosis (Munkonda et al., 2018).
	Exosomes	Islet-derived mesenchymal stem cells (Rahman et al., 2014) and mesangial cells (Barutta et al., 2013).	Unclear (Rahman et al., 2014; Wang X. et al., 2014) and hyperglycemia (Barutta et al., 2013).	Autoimmunity (Rahman et al., 2014).
*Type 2 diabetes*	Microparticles	Endothelial cells (Feng et al., 2010; Tramontano et al., 2010; Jansen et al., 2013a), platelets, monocytes (Nomura et al., 2009), and adipocytes (Eguchi et al., 2015).	Apoptosis (Tramontano et al., 2010) and hyperglycemia (Jansen et al., 2013a).	Endothelial dysfunction (Feng et al., 2010) and procoagulant effect (Sabatier et al., 2002).
	Exosomes	Cardiomyocytes (Wang X. et al., 2014; de Gonzalo-Calvo et al., 2017), and CD34^+^ PBMNC (Mocharla et al., 2013).	Unclear (Wang X. et al., 2014), exposure to elevated VLDL and IDL (de Gonzalo-Calvo et al., 2017), and hyperglycemia (Mocharla et al., 2013).	Anti-angiogenesis (Wang X. et al., 2014) and impaired proangiogenic effects (Mocharla et al., 2013).
*Hypertension*	Microparticles	Platelets, endothelial cells, and monocytes (Preston et al., 2003; Nomura et al., 2009; Cordazzo et al., 2013).	Elevated systolic and diastolic pressure (Preston et al., 2003) and Angiotensin II (Cordazzo et al., 2013).	Procoagulant effect (Cordazzo et al., 2013).
	Exosomes	Cardiomyocytes (Pironti et al., 2015) and macrophages (Osada-Oka et al., 2017).	Mechanical stretching (Pironti et al., 2015) and Angiotensin II (Osada-Oka et al., 2017).	AT1R transfer (Pironti et al., 2015).
*Sleep apnea syndrome (intermittent hypoxia)*	Microparticles	Platelets (Bikov et al., 2017).	Hypoxia (Bikov et al., 2017).	Procoagulant effect (Bikov et al., 2017).
	Exosomes	Endothelial cells (Khalyfa et al., 2016).	Hypoxia (Khalyfa et al., 2016).	Increased permeability and endothelial cell dysfunction (Khalyfa et al., 2016).
*Atherosclerosis*	Microparticles	Endothelial cells (Buendia et al., 2015), SMC (Leroyer et al., 2007), leukocytes (Chironi et al., 2006), and platelets (Leroyer et al., 2007).	TNFα (Heathfield et al., 2013; Buendia et al., 2015) and oxidized LDL (Fu et al., 2017).	Smooth muscle cell pro-calcificant activity (Buendia et al., 2015), thrombogenic effect (Leroyer et al., 2007), and endothelial dysfunction (Brodsky et al., 2004; Fu et al., 2017).
	Exosomes	Visceral adipose tissue-derived adipocytes (Xie et al., 2018), platelets (Srikanthan et al., 2014), CD4+ T cell (Zakharova et al., 2007), mature DCs (Gao et al., 2016), endothelial cells (Zhan et al., 2009), and vascular SMC (Kapustin et al., 2015).	High fat diet (Xie et al., 2018), activated platelets (Srikanthan et al., 2014), T cell activation (Zakharova et al., 2007), DC maturation (Gao et al., 2016), oxidized LDL and homocysteine (Zhan et al., 2009), and increased extracellular calcium, PDGF-BB, TNFα (Kapustin et al., 2015).	Foam cell generation and M1 phenotype macrophage transition (Xie et al., 2018), reduced expression of scavenger receptor CD36 (Srikanthan et al., 2014), TNFα stimulation (Gao et al., 2016), cholesterol accumulation in monocytes and TNFα secretion (Zakharova et al., 2007),monocyte activation via TLR4 (Zhan et al., 2009), and vascular calcification (Kapustin et al., 2015).
*Plaque rupture and acute thrombosis*	Microparticles	Endothelial cells (Schiro et al., 2015), leukocytes (Mallat et al., 1999; Rautou et al., 2011; Sarlon-Bartoli et al., 2013), and SMC, platelets (Rautou et al., 2011).	Apoptosis (Mallat et al., 1999) and cell activation (Rautou et al., 2011).	Endothelial cell apoptosis, matrix degradation, neoangiogenesis, and procoagulant activity (Rautou et al., 2011).
	Exosomes	Platelets (Tan et al., 2016) and SMC (Kapustin and Shanahan, 2016).	Thrombin activation (Tan et al., 2016) and SMC activation (Kapustin and Shanahan, 2016).	Inhibition of PDGFRβ expression on SMC (Tan et al., 2016) and thrombosis (Kapustin and Shanahan, 2016).
*Acute myocardial damage*	Microparticles	Endothelial cells (Stepien et al., 2012; Zhang Y. et al., 2017), platelets, leukocytes, and monocytes (Stepien et al., 2012).	Ischemia (Stepien et al., 2012; Zhang Y. et al., 2017) and platelet activation (Stepien et al., 2012).	Endothelial dysfunction (Zhang Y. et al., 2017).
	Exosomes	Cardiomyocytes (Yang et al., 2016) and pericardium (Foglio et al., 2015)	Ischemia (Foglio et al., 2015; Yang et al., 2016)	Inhibition of autophagy (Yang et al., 2016), angiogenesis and cardioprotection (Foglio et al., 2015)


#### Atherosclerosis

Concerning atherosclerosis, a large body of literature has shown the involvement of EV in crucial steps of its natural history. Endothelial cell dysfunction, the earliest event in the natural history of the disease can be promoted by endothelial derived microparticles. Indeed, early reports showed that MVs released by cultured endothelial cells reduce the acetylcholine induced vasorelaxation of isolated aortic rings, increasing the production of superoxide in target endothelial cells (Brodsky et al., 2004). More recently, microparticles were isolated both from the peripheral blood and atherosclerotic plaques of patients undergoing carotid endarterectomy. In both cases, most of the microparticles were of leukocyte origin. However, while a large fraction of circulating EV were of platelet origin, ≈13% of the vesicles isolated from the plaque were originated from SMC. Functionally, microparticles isolated from both sources expressed similar levels of tissue factor, but those of plaque origin generated more thrombin *in vitro* (Leroyer et al., 2007). Concerning atherosclerosis promoting factors, oxidized LDL and inflammatory mediators (e.g., TNFα) were shown to induce the release of microparticles from a broad range of cells, including endothelial cells, SMC, leukocytes, and platelets (Chironi et al., 2006; Leroyer et al., 2007; Heathfield et al., 2013; Buendia et al., 2015; Fu et al., 2017). Specifically, endothelial cells exposed to TNFα *in vitro* released microparticles that contained high amounts of BMP2 triggering vascular smooth muscle cell (SMC) calcification. Intriguingly, an identical procalcificant mechanism characterized MVs either released by senescent endothelial cells or isolated from patients affected by chronic kidney disease (Buendia et al., 2015). Similarly, stimulation of endothelial cells with oxidized LDL promoted the release of microparticles expressing ICAM-1 that could transfer this molecule to target endothelial cells, increasing their adhesion to monocytes (Fu et al., 2017).

Exosomes too have been implicated in the pathogenesis of atherosclerosis. Specifically, it has been shown that activated CD4^+^ T cells release exosomes that stimulated, *in vitro*, the accumulation of neutral lipids and free cholesterol, in THP-1 derived monocytes. Noteworthy, exosome-stimulated monocytes secreted TNFα as well (Zakharova et al., 2007). Furthermore, endothelial cells exposed to oxidized LDL or homocysteine released exosomes containing HSP70, which, in turn, increased the adhesion of monocytes to endothelial cells (Zhan et al., 2009). Exosomes derived from cultured, mature DCs may also represent an inflammatory stimulus for endothelial cells. Indeed, TNFα, exposed on the surface of these DC-derived particles, triggered, in target human umbilical vein endothelial cells, NFkB activation and expression of adhesion molecules (i.e., VCAM-1 and ICAM-1). Moreover, the *in vivo* administration of DC-derived particles to *ApoE^-/-^* mice worsened the atherosclerotic lesions of the animals (Gao et al., 2016). Cultured SMC too release exosomes in their supernatant, in a nSMase 2 dependent fashion. The release of SMC-derived exosomes was upregulated by calcifying conditions (i.e., in the presence of elevated levels of calcium and phosphate) and by PDGF-BB, while the proteomic analysis of these particles suggested their involvement in calcification processes. Consistently, SMC-derived exosomes could be detected in calcified arteries (Kapustin et al., 2015). Adipocyte derived exosomes too play a role in the atherogenic process. Specifically, visceral adipose tissue derived particles promoted cholesterol accumulation and enhanced macrophage foam cell formation by downregulating ATP-binding cassette transporter mediated cholesterol efflux (via ABCA1 and ABCG1) in RAW264.7 macrophages. Moreover, exosomes released by visceral adipocytes of animals fed on a high fat diet (VAT-HFD) induced the acquisition of a M1 phenotype by Raw264.7 macrophages. This process was a consequence of the activation of NFkB in macrophages and was paralleled by the increased secretion of proinflammatory cytokines IL6 and TNFα. *In vivo* administration of VAT-HFD derived exosomes too could exacerbate the atherosclerotic lesions of *ApoE^-/-^* mice (Xie et al., 2018). Last, exosomes may also have a protective effect, as in the case of those that are platelet derived and are produced during the atherothrombotic processes. Indeed, these particles promoted the ubiquitination of CD36, thus inhibiting both platelet activation and oxidized LDL binding to macrophages *in vitro* (Srikanthan et al., 2014).

Extracellular vesicles are involved in plaque rupture. Specifically, microparticles released by endothelial cells, leukocytes, SMC, and platelets can promote endothelial cell apoptosis, matrix degradation, inflammation, and intra plaque neoangiogenesis, thus destabilizing the plaque and increasing thrombus formation (Mallat et al., 1999; Rautou et al., 2011; Sarlon-Bartoli et al., 2013; Schiro et al., 2015). The clinical relevance of this phenomenon is supported by the observation that higher levels of circulating endothelial microparticles could be observed in the bloodstream of asymptomatic patients with unstable carotid artery plaques (Schiro et al., 2015). Moreover, the levels of circulating of CD11b^+^CD66b^+^ leukocyte derived microparticles proved to be independent predictors of carotid artery plaque instability (Sarlon-Bartoli et al., 2013).

Exosomes too could be produced by platelets and SMC in the context of a vulnerable plaque, contributing to the pathophysiology of the atherosclerotic plaque by favoring calcification and thrombosis (Kapustin and Shanahan, 2016; Tan et al., 2016).

#### Myocardial Infarction

With regard to ST-elevation myocardial infarction (STEMI), circulating microparticle levels increase acutely after the event and are associated with microvascular obstruction (Suades et al., 2016). Indeed, both ischemia and platelet activation trigger the release of MVs by endothelial cells, platelets, leukocytes, and monocytes (Stepien et al., 2012; Suades et al., 2016; Zhang Y. et al., 2017). Importantly, the levels of endothelial and platelet microparticles correlated with the dimension of the area at risk, in STEMI patients (Jung et al., 2012). Functionally, circulating microparticles isolated from infarcted patients were endowed with procoagulant activity, and altered endothelium-dependent vasorelaxation by impairing the endothelial nitric oxide pathway (Boulanger et al., 2001; Morel et al., 2004).

Concerning exosomes, their levels increase in the plasma of patients affected by both myocardial infarction and unstable angina, few hours after the acute event. Consistently, H9c2 myocytes released, *in vitro*, exosomes in their culture medium and the process was enhanced by hypoxia. Importantly, the HIF1α target microRNA30a that is released within exosomes, peaked after 4 h of hypoxia, was efficiently transferred to target myocytes, inhibited autophagy, and promoted apoptosis (Yang et al., 2016). Exosomes may also be found in the pericardial fluid (PF) and their concentration increases after acute myocardial infarction. These EV contain clusterin, that, in turn, promoted the epithelial to mesenchymal transition of epicardial cells, increasing the frequency of epicardial cells coexpressing smooth muscle actin and the stem cell marker c-Kit. Importantly, the *in vivo* administration of clusterin to infarcted mice increased angiogenesis and ameliorated myocardial function (Foglio et al., 2015). In line with these data, the direct proangiogenic effect of exosomes collected from the PF has been recently shown. Specifically, authors have shown the ability of PF derived exosomes to restore the angiogenic capacity of endothelial cells deprived of their microRNA content via Dicer silencing. Furthermore, PF exosomes improved post-ischemic blood flow recovery, in a mouse limb ischemia model (Beltrami et al., 2017).

#### Diabetes

In diabetes, different stimuli, such as hyperglycemia and apoptosis, promote the release of MVs from endothelial cells, platelets, leukocytes, SMCs, mesangial cells, and podocytes (Tramontano et al., 2010; Barutta et al., 2013; Jansen et al., 2013a; Chiva-Blanch et al., 2016; Ishida et al., 2016; Lytvyn et al., 2017; Munkonda et al., 2018). Functionally, microparticles isolated from diabetic rat plasma that originate mainly from platelets, were able to decrease acetylcholine-induced endothelium-dependent relaxation on carotid artery rings, *ex vivo*. These effects were associated with a decreased expression of eNOS, and with an incremented expression of caveolin-1 in target carotids (Ishida et al., 2016). Microparticles released by endothelial cells exposed to hyperglycemia were characterized by increased NADPH oxidase activity and reactive oxygen (ROS) levels. When human coronary endothelial cells were cultured in the presence of these pathologic microparticles, they increased intracellular ROS levels, activated p38 MAPK in a ROS dependent fashion, and expressed the adhesive proteins ICAM-1 and VCAM-1, thus incrementing monocyte adhesion (Jansen et al., 2013a). Microparticles released by endothelial cells and monocytes seem to link vascular injury with kidney disease. Indeed, addition of microparticles released by these two cell types, in response to TNFα stimulation, to differentiated podocytes upregulated the production of the proinflammatory mediators MCP-1 and interleukin-6, *in vitro* (Eyre et al., 2011). In turn, proximal tubule epithelial cells, cultured in the presence of podocyte derived microparticles, activated p38 MAPK, and TGFb signaling, promoting the secretion of fibronectin/collagen type IV, potentially contributing to renal fibrosis. This effect could be inhibited blocking the scavenger receptor CD36 (Munkonda et al., 2018). Last, Annexin V^+^ microparticles can be released by adipocytes during lipotoxic stress, in a Caspase-3 and Rho-associated kinase fashion, promoting migration of monocytes and macrophages to the adipose tissue (Eguchi et al., 2015).

Concerning exosomes, it has been shown that they could play a relevant role in the pathogenesis of type 1 diabetes. Indeed, islet-derived mesenchymal stem cells (iMSC) released exosomes that were able to activate autoreactive B and T cells, primed in NOD mice. Immunization of mice with iMSC derived exosomes favored the expansion of diabetogenic T cells and accelerated the T cell mediated destruction of islets (Rahman et al., 2014). Although it has been shown that exosomes and specific microRNAs could be found in the urine during the evolution of diabetic nephropathy, few works have demonstrated the direct pathophysiological role of these small EV (Barutta et al., 2013). However, it was recently shown that rat proximal tubule cells exposed to advanced glycation end-products (AGE) increased the secretion of exosomes containing C-megalin. The latter is a multiligand endocytic scavenger receptor that binds to ligands such as AGE-modified bovine serum albumin, promoting dysfunction of the autophagy/lysosomal pathway and exosomal C-megalin excretion (De et al., 2017). Hyperglycemia exerts also a negative impact on CD34^+^CD14^+^ and CD34^+^CD14^-^ circulating proangiogenic cells by inhibiting the secretion of the angiogenic microRNA126 within exosomes (Mocharla et al., 2013). Last, cardiomyocytes too, when exposed to hyperglycemia or VLDL/IDL, secrete exosomes (Wang X. et al., 2014; de Gonzalo-Calvo et al., 2017). These contain microRNA1 and microRNA133a and proved to be independent predictors of myocardial steatosis, in uncomplicated type-2 diabetes (de Gonzalo-Calvo et al., 2017). Functionally, exosomes released from cardiomyocytes isolated from Goto-Kakizaki rats (an animal model that spontaneously develops type 2 diabetes) inhibited the activities of proliferation, migration, and formation of tubule-like structures of cardiac endothelial cells. The effect was mediated by the transfer of the antiangiogenic microRNA320 to target cells (Wang X. et al., 2014).

Last, diabetes, may reduce the positive effect exerted by EV. Indeed, although endothelial microparticles can promote angiogenesis via microRNA126, diabetes reduces the microRNA126 content of these EV, thus impairing their proangiogenic capability (Jansen et al., 2013b). Similarly, while cardiomyocytes release exosomes that promote endothelial cell proliferation, migration, and capillary network formation *in vitro*, diabetes abrogated this protective effect (Wang X. et al., 2014).

#### Hypertension

With regard to other risk factors for cardiovascular disease, both elevated artery blood pressure and angiotensin II (Ang II) stimulate the release of microparticles from platelets, endothelial cells, and monocytes (Preston et al., 2003; Nomura et al., 2009; Cordazzo et al., 2013). Importantly, the levels of both platelet and endothelial microparticles correlated with: blood pressure, markers of endothelial and platelet activation (i.e., sVCAM-1 and sICAM-1), and vWF, a marker of endothelial injury/dysfunction (Preston et al., 2003). In a cohort of hypertensive patients with diabetes, systolic blood pressure, HDL cholesterol, sP-selectin, sE-selectin, sCD40L, RANTES, monocyte derived microparticle levels, and endothelial derived microparticle levels were independent predictors of the levels of platelet derived microparticles (Nomura et al., 2009). These results suggest that microparticles are at the crossroads between endothelial injury, coagulation, and inflammation. In line, exposure of mononuclear cells to Ang II, increased intracellular calcium levels and stimulated the release of procoagulant, tissue factor-bearing MVs (Cordazzo et al., 2013). Similarly, stimulation of endothelial cells with Ang II promoted superoxide generation, Rho kinase activity, and MV release, which, in turn, exerted prooxidative and proinflammatory effects on normal endothelial cells (Burger et al., 2011). Importantly, Ang II receptor blockers inhibited MV release.

Concerning exosomes, both mechanical stretching, and Ang II can promote their release from cardiomyocytes and macrophages (Pironti et al., 2015; Osada-Oka et al., 2017). Functionally, these vesicles could mediate the transfer of AT1R on cardiac and skeletal myocytes, and mesenteric vessels, conferring responsiveness to Ang II infusion in AT1R knockout mice (Pironti et al., 2015). Moreover, THP-1-derived macrophages, grown in the presence of Ang II, released exosomes that increased the expression of ICAM-1 and PAI-1 on target endothelial cells (Osada-Oka et al., 2017).

#### Sleep Apnea Syndrome and Hypoxic Conditions

In patients suffering from sleep apnea syndrome, episodes of intermittent hypoxia are associated with the release of circulating CD41^+^ and Annexin V^+^ microparticles of platelet origin, which show a diurnal fluctuation that reaches a significant peak at 5 pm. While the levels of CD41^+^ MVs positively correlated with the severity of obstructive sleep apnea, continuous positive airway pressure therapy reduced their diurnal peak (Bikov et al., 2017). Moreover, exposure of healthy volunteers to intermittent hypoxia led to the release of exosomes of endothelial cell, lymphocyte, monocyte and platelet origin into the bloodstream. Exosomes released after 4 days of exposure to intermittent hypoxia (4D IH) increased the expression of ICAM-1, decreased the expression of eNOS, and reduced the cell barrier function of cultured endothelial cells. Moreover, endothelial cells treated with 4D IH exosomes increased their adhesion to monocytes (Khalyfa et al., 2016). Indeed, hypoxia is a potent trigger for EV secretion, activating HIF1α and HIF2α (Wang T. et al., 2014). Furthermore, during ischemia reperfusion injury, stimuli such as ROS and activation of purinergic receptors can act as additional triggers of EV release (Szotowski et al., 2007; Kahner et al., 2008; Bianco et al., 2009; Drago et al., 2017).

### Effect of Therapies on EV Release

Considering the biogenesis and the mechanisms of release of EV, it is conceivable how not only diseases but also drugs can impact EV production.

Since variations in intracellular calcium levels modulate the release of both exosomes and MV, we can anticipate that drugs interfering with calcium homeostasis modify the secretory profile of treated cells. In line, amiloride, a drug that inhibits both the Na^+^/H^+^ exchanger and the Na^+^/Ca^2+^ channel, has been shown to reduce both constitutive and stimulated exosome release *in vitro* (Savina et al., 2003). Similarly, nifedipine, a calcium channel blocker, was able to reduce the release of platelet derived microparticles (Lee et al., 1993). However, the effect of the latter drug on the release of EV by cardiac cells has not been assessed yet. Conversely, no effect was observed when the L-type calcium channel inhibitor verapamil was employed (Savina et al., 2003). Importantly, calcium channel blockers and β-blockers may also inhibit EV uptake, by reducing endothelial cell activation (Xiao X. et al., 2017).

An intriguing observation, conducted in the oncology field, is that a low environmental pH is associated with enhanced secretion and uptake of exosomes (Parolini et al., 2009). In line, omeprazole and other H^+^/K^+^ pump inhibitors, such as omeprazole, reduce both exosome release and their uptake (Chalmin et al., 2010). Although these observations have been conducted on tumors, it would be of outmost important to verify whether these drugs interfere with EV release during cardiac ischemic events, conditions that are coupled with the acidification of the interstitial space.

Consistently with the role played by cholesterol in exosome biogenesis, it has been shown that hydroxy-methylglutaryl-coenzyme A (HMG-CoA) inhibitors modulate the release of exosomes. Specifically, they increase exosome secretion from microglial cells, and promote the release of immunomodulatory exosomes from DCs (Tamboli et al., 2010; Li et al., 2016). Concerning other drugs interfering with lipid metabolism, it has been shown that amiloride could inhibit ceramide formation, possibly by indirectly inhibiting the acid sphingomyelinase, preventing the formation of an acidic microenvironment (Serrano et al., 2012). Similarly, GW4869, a nSMase 2 inhibitor, could blunt ceramide formation and exosome biogenesis (Panigrahi et al., 2018).

Given the described role played by ADP in promoting EV release, it is not surprising that thienopyridines, e.g., ticlopidine, clopidogrel, and prasugrel (P2Y_12_ inhibitors), can negatively modulate the release of EV (Badimon et al., 2017). Furthermore, other antiaggregant drugs, such as aspirin, lower the level of circulating microparticles both *in vitro* and in diabetic patients (Chiva-Blanch et al., 2016; Connor et al., 2016).

In line with the stimulatory effect of TNFα on the release of endothelial cell derived MVs (see **Table 1**), the use of an inhibitor of this cytokine (certolizumab) reduced EV secretion *in vitro* (Heathfield et al., 2013).

Moreover, while digoxin possibly reduces exosome release, acting as a non-specific HIF1α inhibitor, it has been shown to increase the release of endothelial cell and platelet derived microparticles, in patients affected by atrial fibrillation (Chirinos et al., 2005; Panigrahi et al., 2018).

Of particular relevance in oncology is that cisplatin, which is usually concentrated in the lysosomal compartment, could be secreted via the exosomal pathway, in drug resistant cells (Safaei et al., 2005). Similarly, doxorubicin can be secreted within exosomes, but this process could be inhibited *in vitro* by ketotifen, a stabilizer of the mastocyte membrane, thus reversing drug resistance (Khan et al., 2018).

Last, prompted by early reports, showing that the cardioprotective and proangiogenic activities of both mesenchymal stem cells and CD34^+^ progenitors could be found in the exosomal fraction of their secretome, several investigators have evaluated, in recent years, the cardioprotective role of EV secreted by different cell types (Lai et al., 2010; Sahoo et al., 2011). In line, microparticles released by apoptotic endothelial cells promote endothelial cell migration and proliferation, thus promoting reendothelialization *in vivo*, with a microRNA126-dependent mechanism (Jansen et al., 2013b). Cardiac progenitors (CPC) too release exosomes that are required to protect HL-1 myocytes from apoptosis and to promote angiogenesis *in vitro*. These exosomes, that contain proangiogenic and anti-apoptotic microRNAs, reduced the infarct size, decreasing cardiomyocyte apoptosis, and increasing angiogenesis *in vivo* (Barile et al., 2014). Intriguingly, the protective effect of CPC-derived exosomes mimicked the effect of cell therapy on acute myocardial infarction, possibly as a consequence of the modulation of the immune system. Specifically, exosomes reduced the accumulation of macrophages in the zone bordering the infarcted area and promoted their polarization toward an anti-inflammatory phenotype, both *in vivo* and *in vitro*. Exosome-mediated transfer of microRNA181b to macrophages may be the molecular mechanism responsible for the phenomenon (de Couto et al., 2017). The protein content of exosome too has been considered to be responsible for the beneficial effects of CPC-derived exosomes. Indeed, pregnancy-associated plasma protein-A (PAPP-A) is one of the most highly enriched proteins in the comparison between CPC-derived exosomes and bone marrow mononuclear cell derived exosomes. PAPP-A, whose active form was identified on the exosome surface, cleaves IGFBP-4 and increases the bioavailability of IGF-1, thus reducing cardiomyocyte apoptosis. Consistently, CPC-derived exosomes reduced infarct size and ameliorate ventricular function more efficiently than bone marrow derived ones (Barile et al., 2018).

## Conclusion

In conclusion, the term EV encompasses a broad category of cell derived vesicles that differ in biogenesis and biological properties. As a consequence, their fractionation and characterization still represent a challenge. As consequence of this and of the fact that it is an emerging area of investigation, the current literature may be confounding. However, the conflicting results could just reflect heterogeneity in EV preparation and characterization and lack of knowledge or attention to confounding factors. Among the most prominent ones, comorbidities and ongoing pharmacological therapies may have a profound impact in either stimulating or inhibiting EV release or in modifying the characteristics of the secreted EV. In line, according to the literature, EV secreted by a specific cell type may exert protective effects, under normal conditions, while they could exert deleterious effects in pathological settings. Similarly, priming of a cell type with a specific drug may increase the immunomodulatory function of EV secreted by a given cell type.

Therefore, in future studies, the pathologic status, and the ongoing drug therapies must be considered as covariates, when analyzing for the biological effect of a specific EV type.

## Methods

To identify the scientific literature regarding EV and cardiovascular diseases, we reviewed the literature in the PubMed database. We focused on publications written in English and published in the last ≈10 years. As search terms, we used “EV” or “MVs” or “exosomes” and either “cardiovascular disease” or “diabetes”. Due to the large number of papers, more than 2,000 scientific works, we were not able to cite all individual references and we summarized those that we consider the most important concepts and quoted representative works. We apologize to all authors whose important publications are not cited.

## Author Contributions

AA, DC, and AB wrote and reviewed the manuscript. PP, CC, CV, and AZ reviewed the manuscript.

## Conflict of Interest Statement

The authors declare that the research was conducted in the absence of any commercial or financial relationships that could be construed as a potential conflict of interest.
